# Thermal Stability, Blocking Regime and Superparamagnetic Behavior in Mn-Al-C Melt Spun Ribbons

**DOI:** 10.3390/nano11112898

**Published:** 2021-10-29

**Authors:** Alina Daniela Crisan, Aurel Leca, Ioan Dan, Ovidiu Crisan

**Affiliations:** 1 National Institute for Materials Physics, P.O. Box MG-7, 077125 Magurele, Romania; ad_crisan@yahoo.com (A.D.C.); aurel.leca@infim.ro (A.L.); 2 R & D Consulting and Services S.R.L., 023761 Bucharest, Romania; ioan_dan@rd-consultanta.ro

**Keywords:** MnAl systems, ε–τ phase transformation, magnetic properties, magnetic transitions

## Abstract

Alloys possessing nominal compositions Mn_53_Al_45_C_2_ and Mn_52_Al_46_C_2_ were prepared by the melt spinning method and were subjected to complex structural, morphological and magnetic investigations. As these alloys can exhibit tetragonal L1_0_-type and τ phase, they have good potential as rare earth (RE)—free magnets. It is, therefore, important to monitor the ε–τ phase transformation and the stability and the magnetic features of the tetragonal phase in an entire temperature interval. By using synchrotron X-ray diffraction, it has been proven that the ε–τ phase transformation occurs gradually, with the τ phase becoming predominant only after 450 °C. Moreover, this phase has been proven to be quite stable without any grain growth even at the highest temperature investigated at 800 °C. Low temperature behavior was thoroughly investigated by using a complex combination of major and minor hysteresis loops combined with the zero field cooled-field cooled magnetization protocols (ZFC-FC). Two different regimes, blocking and superparamagnetic, were documented. A spin reorientation transition was proven to occur at 55 K while a maximum magnetization observed in ZFC-FC curves proved that at about 75 K, a transition from ferro to superparamagnetic state occurs. The existence of a blocking regime below 55 K that is characteristic to nanogranular systems with superparamagnetic behavior has shown further development towards obtaining RE-free magnets.

## 1. Introduction

As an intermetallic binary alloy, Mn-Al has attracted a long lived interest ever since the work of H. Kono et al. [1]. As a potential breakthrough solution for the low cost, rare earth free magnetic materials, the Mn-Al system has attracted a growing interest only recently, expressed by an ever growing number of publications [2,3,4,5,6,7,8,9,10]. It is known that MnAl alloy upon annealing can become ferromagnetic in certain conditions depending on the relative stoichiometry of the two elements. Proceeding further, a third alloying element has been added in a number of scientific works in order to increase either its degree of ordering and refine the alloy microstructure or the overall magnetic properties. Mn-Al has certain potential in developing reasonable coercive fields and large specific magnetization. It is also worth mentioning that Mn-Al could develop large anisotropic fields [2,3,4,5,6] and may be of interest for technologic applications due to their low cost, high abundance of the raw materials, resilience, corrosion-effective resistance and also being easily processed, since they do not show brittleness as is the case of several Fe-based soft magnets. The face-centered-tetragonal *fct* τ-phase is responsible for strong anisotropic fields and overall magnetic performance of the Mn-Al magnets, with close similarities to the *fct* L1_0_ phases, which are phases that have been obtained in both FePt and CoPt melt spun alloys [7,8] with strong uniaxial magnetocrystalline anisotropy [9]. To actually produce a Mn-Al-based magnet with noticeable overall magnetic performances is, however, extremely difficult [10] since τ-phase is structurally metastable. In addition to that, the τ-phase is only obtainable in a very small compositional interval. Several publications reported that *fct* τ-MnAl emerges from the precursor hexagonal ε-phase for compositions between 51 and 58 at.% Mn [11,12], after thermal treatment above 650 °C. In thermodynamic equilibrium [13] an abundance of 51 up to 58 at.% Mn provides an alloy where both γ_2_-Mn_5_Al_8_ and the Al solid solution in β-Mn coexist. It has to be mentioned that, if τ-MnAl is fully ordered, Mn has (0,0,0) occupancy for the atom positions while Al atoms occupy the (½,½,½) crystallographic positions. As most of the times the alloy is constructed to be slightly off equiatomic composition, excess Mn occupies some of the Al (½,½,½) sites. This produces an increased amount of antiferromagnetically aligned Mn moments and, hence, will decrease the alloy’s magnetization. In one of the seminal publications on Mn-Al [14], a maximum magnetization is obtained for 51 at.% Mn, but this value diminishes if Mn concentration is increased. It has also been shown that adding carbon to the binary alloy increases, furthermore, the magnetization.

Stoichiometry not only plays a role in forming the τ–MnAl, but microstructural arrangements of grains also play a role upon casting the alloy and the thermodynamic equilibrium. The similarity of the tetragonal τ-phase with the superlattice features of other L1_0_ phases has also been reported [15,16,17,18,19,20]. Two main pathways for obtaining τ–MnAl have been reported [21,22]: (a) ultra-rapid quench of equilibrium ε-phase successively followed by thermal treatment and (b) slow cooling of the parent disordered ε-phase. Proving evidence has been reported that τ–MnAl emerge from annealing hexagonal ε–MnAl at about 500 °C; however, this may produce some structural instabilities [23]. The Mn-Al phase diagram [13] shows that, indeed, τ–MnAl phase is bound to decompose into β-(Mn) and γ_2_ (Mn_5_Al_8_) for temperatures surpassing 650 °C. To avoid this decomposition and to preserve its stability, one main pathway is represented by the addition of a few atomic percentages of carbon to the binary Mn-Al alloy [4]. It has been shown [3] that the Mn-Al-C samples sintered at 550 °C under a uniaxial pressure of 100 to 400 MPa exhibit not only good anisotropy of magnetic properties but also strong dependence of the coercivity on stress induced through sintering. On the other hand, it has been shown [2] that magnetic properties of MnAl were sensitive to the C doping, and the addition of 2% and 3% C directly gave rise to the ferromagnetic τ phase in the as-melted MnAl–C.

The phase stability in MnAl alloys has been extensively studied also by theoretical means. It has been found through ab initio calculations that ferromagnetism may be found in the ground state of the τ–MnAl [24,25,26] with an overall magnetic moment of as much as 160 emu/g. Some large values of anisotropy energy density (1 MJ/m^3^) have been predicted through density functional theory calculations. Density functional theory approaches [27] showed that large magnetocrystalline anisotropies of about 1 MJ/m^3^ (or 12.5 MGOe) can be expected as well as 0.8 MA/m (about 1 kG) saturation (volume) magnetization. Curie temperature of Mn-rich side of the alloys was estimated at 600 K [27] by using Monte Carlo simulations. The occurrence of the τ-MnAl phase was argued to be strongly dependent on the stoichiometry of the initial chemical composition, synthesis methods and subsequent thermal treatment. Evidence was brought about with respect to the fact that the phase transformation of Mn-Al from ε-phase to τ-phase is a two-step process [28,29,30]. In many cases, whatever the preparation pathway, mixtures of various phases are observed rather than only single phase. In the research described in [29,31,32], the microstructure is made by τ-phase coexisting with ε-phase. Then, in [33,34], a coexistence of γ_2_, β and τ phases is proven, while in the latest example [35], τ, β and ε-phases are found co-existing at the same time. From other various reports [36,37,38], it was observed that τ-phase comes from slowly cooling the melt spun ribbon to ambient temperature. There is a method to avoid splitting into β-Mn and γ_2_ (Mn_5_Al_8_); the parent ε-MnAl phase has to be cooled very quickly [39,40,41]. Mitsui et al. [42] used in-magnetic-field annealing to investigate Zn-modified Mn-Al and Mn-Al-C up to 573 K. It was found that magnetic field annealing was beneficial for the Zn-modified Mn-Al but detrimental for C-modified Mn-Al in what concerns stabilization of τ-phase against ε-phase. Dehghan and Ebrahimi [43] have also studied the effect of strain on the magnetic properties of Mn-Al-C samples that are hot compressed, and it was found that by increasing strain and strain rate, the coercivity increases, and higher remanent magnetization was obtained in axial direction for lower strains. K.P. Su et al. [44] studied Mn-Al based hard magnetic magnets with C addition, obtained as flakes by surfactant assisted ball milling. Texturing along [001] axes of τ–MnAl phase was observed, and coercivity was found to be at around 2500 Oe even after 15 h milling. In a very recent publication, R. Kobayashi et al. [45] have shown that ferromagnetic Mn–Al–C (τ-phase) can be synthesized by a single-route conventional reactive sintering method. The maximum magnetization and coercivity were 75.8 Am^2^/kg and 57 mT, respectively, when τ-phase fraction was about 81 mass% for Mn_55_Al_45_C_2_ annealed at 1273 K. The results have been interpreted in terms of phase stabilization by carbon addition during ε–τ transformation. Further motivation for magnetic phase evolution in MnAlC is also given by the theoretical studies of magnetic hysteresis of Masrour et al. [46,47].

In order to better understand the effects of carbon addition on the magnetic performances and especially on the signature of the ε–τ phase transformation as manifested in the temperature dependent magnetization measurements, we have synthesized two melt spun alloys having nominal composition Mn_52_Al_46_C_2_ and, respectively, Mn_53_Al_45_C_2_. Crystallography studies allowed the determination of the phase structure in the as-cast alloys, X-ray powder diffraction method was used for that purpose. Scanning electron microscopy has been used for determination of the as-cast ribbons morphology and surface analysis. For the determination of the temperature-dependent magnetic response and monitoring of the ε–τ transformation, a zero field cooled-field cooled magnetic experimentation protocol has been used. The as-obtained magnetic behavior was then correlated to the observed ribbons structure and phase composition. These results permitted following the magnetic evolution of intrinsic properties of the two Mn-Al-C samples and permitted highlighting the existence of a blocking regime and superparamagnetic behavior at low temperatures in these alloys.

## 2. Materials and Methods

The two alloys, Mn_52_Al_46_C_2_ and Mn_53_Al_45_C_2_, have been synthesized in the shape of melt spun ribbons by using a Buehler Melt Spinner (Edmund Buehler GmbH, Bodelshausen, Germany). Precursor metallic alloys were synthesized by using elemental metallic flakes of high purity by induction melting under Ar (pressure 10^−1^ Torr). Alloys were then re-melted 3 times in order to better homogenize the mixture. Following that, 5 g per each sample of the primary alloy were then re-melted in a quartz crucible dotted with round nozzle of 2.5 mm diameter. Melt was then purged through the nozzle by an over pressure gas of about 5.5 × 10^4^ Pa on a copper wheel rotating at about 1300 rot/min (linear speed: 31.2 m/s, corresponding roughly to 10^6^ K/min cooling rate of the molten alloy). This technique allowed the formation of long, homogeneous ribbons that were 2 mm wide and 35 microns in thickness. Scanning electron microscopy images (SEM) were taken using an EVO 50 XVP microscope from Carl Zeiss (Carl Zeiss GmbH, Oberkochen, Germany). The X-ray diffractograms were obtained with a Bruker D8 Advance (Bruker AXS GmbH, Karlsruhe, Germany) machine (X-ray generator with Ge detector) using Cu Kα radiation (λ = 0.154 nm). X-ray diffraction data (XRD) were recorded in θ–2θ geometry between 20° and 90° (in 2θ). Full-profile analysis was performed on all recorded diffractograms by means of MAUD (Materials Analysis Using Diffraction) software (MAUD version 2.99, Luca Luterotti, University of Trento, Italy). A temperature-dependent structural study was performed by using the X04 SA materials science beamline at the Swiss Light Source synchrotron X-ray diffraction facility (Paul Scherrer Institute, Villigen, Switzerland). This facility allows obtaining X-ray diffractograms at various temperatures, ranging from 50 to 800 °C. The zero field cooled-field cooled magnetization protocol as well as the magnetic measurements on the Mn_52_Al_46_C_2_ and Mn_53_Al_45_C_2_ samples were performed using SQUID (Superconducting QUantum Interference Device) using a MPMS (Magnetic Properties Measurement System) from Quantum Design (Quantum Design Europe GmbH, Darmstadt, Germany), a facility that has 10^−11^ A m^2^ resolution, maximum applied field 5.6 × 10^6^ A/m with 80 A/m resolution in the applied field, temperature range between 2 K and 400 K and value stability of 10^−3^ K. The hysteresis loops of both samples were measured in an applied field proceeding up to 4 × 10^6^ A/m (about 5 kOe) and applied parallel to the ribbons plane at 300 K.

## 3. Results

### 3.1. SEM Results

In our previous paper [48], we have demonstrated that partial replacement of Mn with small carbon addition was justified on the one hand by the need of obtaining a microstructure where carbon atoms act as nucleation centres for the formation of ordered L1_0_ type phase in MnAl, and on the other hand to facilitate the occurrence of a magnetic interaction of the super-exchange type, where the exchange interaction between adjoining MnAl ordered grains/regions is intermediated and facilitated by carbon atoms, mostly situated in the interphase boundaries between neighboring ferromagnetic L1_0_ regions/grains.

**Figure 1:**
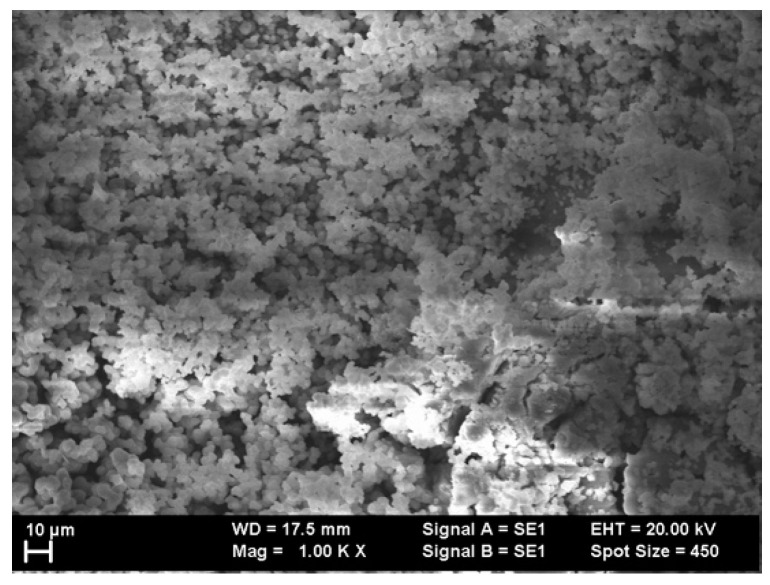
SEM image of Mn_53_Al_45_C_2_ as-cast sample.

**Figure 2:**
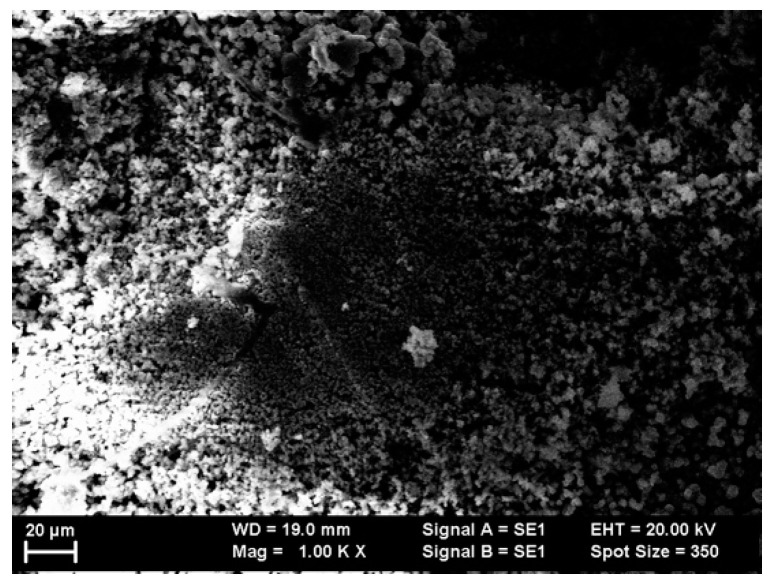
SEM image of Mn_52_Al_46_C_2_ as-cast sample.

In order to further probe the morphology of the as-cast ribbons of Mn_53_Al_45_C_2_ and, respectively, Mn_52_Al_46_C_2_ alloys, scanning probe images were taken on the surface by using the secondary electron module of the SEM microscope (Carl Zeiss GmbH, Oberkochen, Germany). Figure 1 and Figure 2 show two illustrative images of the as-cast samples Mn_52_Al_46_C_2_ and, respectively, Mn_53_Al_45_C_2_.

From these images, one can deduce that at the surface, due to the inhomogeneities in the copper wheel surface, the morphology is rather inhomogeneous, with dendritic growth at the surface and some agglomeration of nanograins with an average size of about 5–10 microns. Moreover, some tendencies of dendritic-type clustering of individual nanograins were observed in the case of Mn_53_Al_45_C_2_ as-cast sample. In the case of the Mn_52_Al_46_C_2_ sample, some small columnar aggregates are visible on the surface; however, the image contrast is consistent over all observed aggregates. This proves that, on one hand, there seems to be no unalloyed metals on the surface, and on the other hand, that all the visible aggregates most probably belong to the same crystalline phase MnAl. These observations come to confirm that there is apparently no multiple phase present in our samples. By using a hystographic method averaging over all the recorded SEM images, for each of the investigated samples, we have derived the grains’ size distribution. We have, thus, observed that the grain size distributions have bimodal character possessing two maxima, and we have calculated that these are centered around 4 ± 0.5 microns and 8 ± 0.7 microns, respectively, for both of the studied alloys.

### 3.2. XRD Results

Structural studies by means of X-ray diffraction have shown [48] that for the two ribbons in the as-cast state, the ε hexagonal phase predominates. This result confirms the hints we have received from the scanning electron microscopy images, i.e., that the two samples are mostly single phased. However, after annealing at 700 °C, the ε phase transforms into the tetragonal τ phase, which becomes predominant.

**Figure 3:**
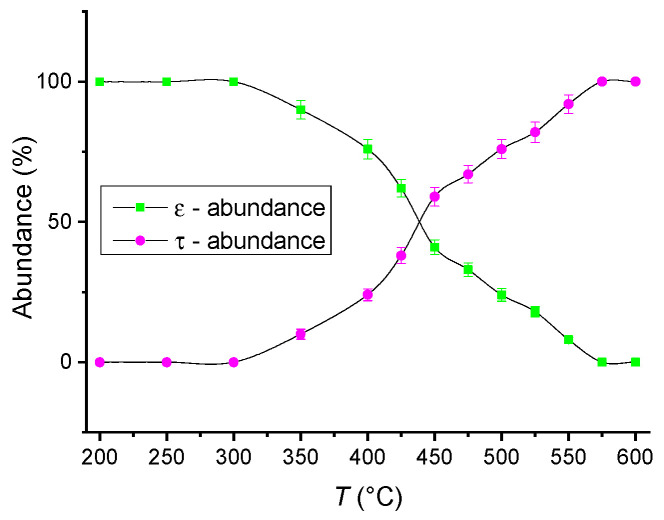
Relative abundance of ε and τ-MnAl, respectively, vs. temperature of the measurement.

**Figure 4:**
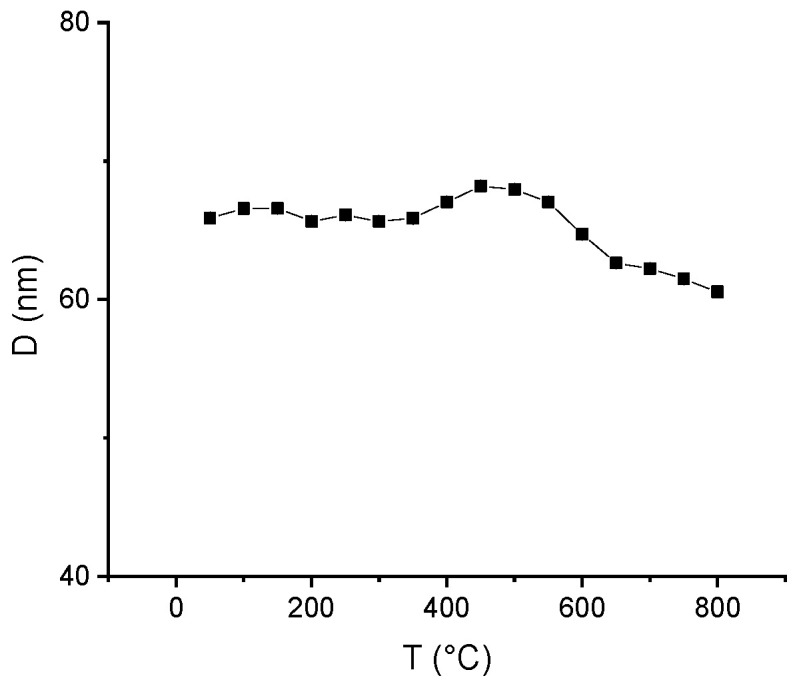
Average crystallite size of Mn_52_Al_46_C_2_ sample vs. temperature of the measurement.

For a better estimation of the structural effects and of the parameters that governs the ε–τ structural phase transition, to determine the path to formation of the tetragonal phase as well as to estimate its stability with the temperature, a temperature-dependent structural study has been undertaken on the two samples. For this purpose, the samples were subjected to measurements using the synchrotron X-ray diffraction facility at the Paul Scherrer Institute, Villigen, Switzerland. This facility allows obtaining X-ray diffractograms at various temperatures ranging from 50 to 800 °C. In our experimental protocol, the samples were measured at temperatures starting from 50 °C until 800 °C in steps of 50 °C for each measurement. The resulting diffractograms have been analyzed using the full-profile Rietveld-type MAUD software. This analysis allowed us to determine both the nature of the crystalline phases observed in the samples and the relative abundances of both ε and τ–MnAl phases during the continuous ε–τ structural phase transition over the investigated temperature range. Figure 3 shows comparatively the relative abundances of the two phases, ε and τ–MnAl, as a function of the temperature of measurement. It can be observed that below the ε–τ transition temperature, the abundance of the ε phase in the Mn_52_Al_46_C_2_ sample is 100%, as this phase is predominant in the as-cast state, until 300 °C. Above this value, the τ–MnAl phase starts to be observed in relatively low proportions (10–25%, until the measurement temperature of about 400 °C). This indicates that the ε–τ structural phase transition is a gradual one, occurring over a large temperature range and at high heating rates, as the ones used during our synchrotron XRD measurement (X04 SA materials science beamline, Swiss Light Source, Paul Scherrer Institute, Villigen, Switzerland) protocol. The abundance of the τ–MnAl phase continues to grow until it becomes equal to the one of the precursor ε phase at about 450 °C. Above this value, the abundance of the τ–MnAl grows further and becomes predominant in such a way that by the end of the analyzed temperature range (700–800 °C), this phase reaches 100% abundance in both the analyzed samples. By our experiment, we have, thus, confirmed that the ε–τ phase transition occurs gradually, a fact which has been reported by other authors as well. Figure 4 shows the temperature dependence of the average crystallite size of τ–MnAl nanograins, a size which was calculated from the full profile analysis of the synchrotron X-ray diffractograms by using the integral breadth method [49]. It is interesting to notice that the average crystallite size for all the investigated temperatures oscillates slightly but is kept at low values between 60.5 and 68.5 nm. It should be noted that the average crystallite size, determined from the full-profile analysis of XRD results, represents mainly the volume-averaged crystallographically coherent domain size or the size of the nanocrystal to the extent where the nanocrystals preserve its lattice structure in the sense given by Balzar [50]. The integral breadth method summarized in [51] calculates the root-mean-square (RMS) strain and both surface-weighted and volume-weighted domain sizes according to the ‘double-Voigt’ method [50,51], which is equivalent to the Warren–Averbach approach [52]. The calculated volume-weighted domain size is not similar to the grain size, as determined by SEM, which in facts quantifies only the aggregates of nanoparticles clustering at the surface and that are visible through SEM imaging. The fact that the average crystallite size does not increase significantly with the increase in temperature, even at the highest investigated temperatures (700–800 °C), brings solid proof that the τ–MnAl has strong structural stability, an issue of high importance when it comes to contemplating potential applications of this material as magnets in extreme conditions of operation. It is, however, worth mentioning that only isothermal annealing can fully stabilize the τ–MnAl phase. We have shown [46] that after isothermal annealing at 700 °C, both Mn-Al-C alloys underwent an irreversible ε–τ phase transformation, and the abundance of the τ phase reaches, in these cases, more than 90%. As a consequence, the magnetic performances are highly improved.

### 3.3. Magnetic Properties

**Figure 5:**
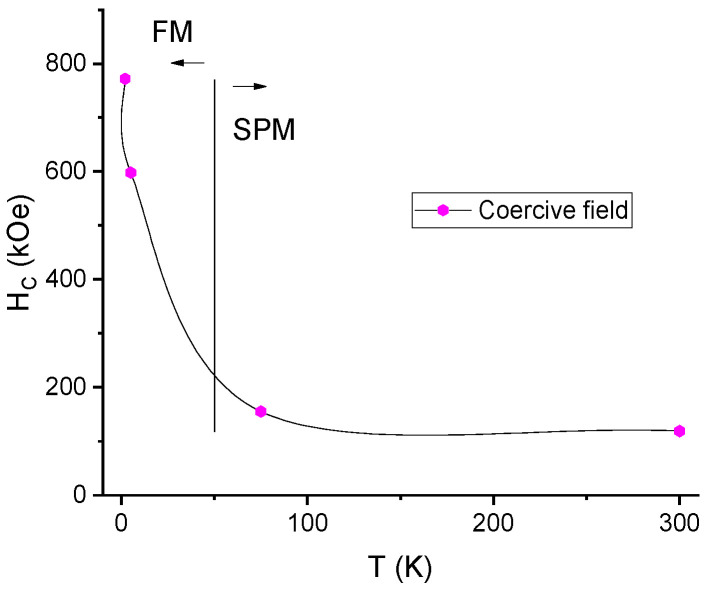
Temperature dependence of the coercive field in Mn_53_Al_45_C_2_ sample. The two magnetic regimes (FM and SPM) are evidenced in the figure.

**Figure 6:**
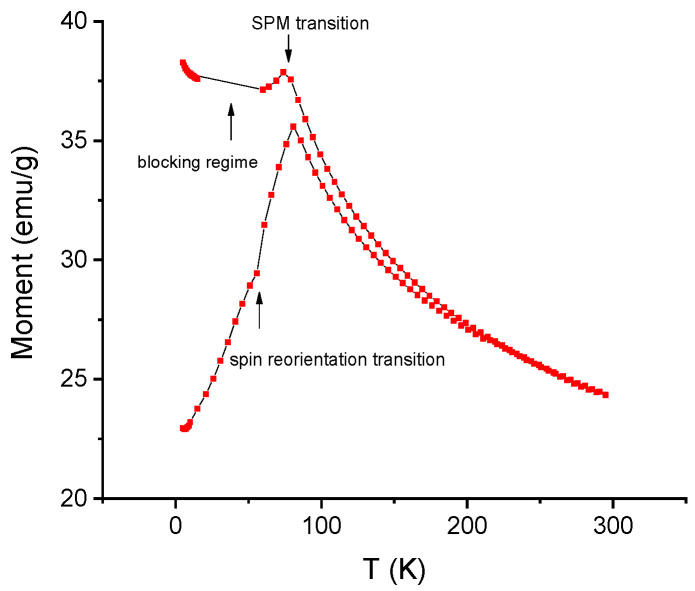
ZFC-FC curves of Mn_53_Al_45_C_2_ sample. The blocking regime and the spin reorientation and SPM transitions are evidenced in the figure.

In order to evaluate the temperature evolution of the magnetic intrinsic properties of the Mn-Al-C alloys, several magnetization measurements have been undertaken. Hysteresis loops have been recorded at various temperatures, ranging from 2 K up to 300 K, with applied magnetic field up to 5 Tesla parallel to the sample plane. The obtained coercive fields from these measurements are plotted in Figure 5 vs. the temperature of the experiment for sample Mn_53_Al_45_C_2_. It can be observed that there is a sharp decrease in coercivity from the maximum value of around 800 Oe measured at 2 K down to around 150 Oe measured at 75 K. Above 75 K, the coercivity remains almost the same, up to the maximum investigated temperature (300 K). This behavior confirms that there are actually two regimes of magnetic performance: the first one with high values of coercivity is associated with a ferromagnetic state of the Mn-Al-C sample, while the second one with low values of the coercive field is associated with the superparamagnetic state of the Mn-Al-C sample. The superparamagnetic state in nanogranular alloys is in fact a size effect; when the thermal barrier (k_B_T) overcomes the anisotropy energy, proportional to the nanoparticle volume (KV), the magnetic moments fluctuate and randomly give rise to low magnetization and low coercivity. Such a state has been documented also in other works; for instance, in polycrystalline MnAlC thin films [53], it has been found that τ-MnAlC films exhibit superparamagnetic-like behavior proven by the shape of the hysteresis loops and the low values of coercivity and magnetization. From this result, a potential ferro–superparamagnetic transition is inferred; for confirming this assumption, a zero field cooled–field cooled (ZFC) magnetization measurement has been performed for the same temperature range. For this experimental protocol, in the first run, the sample has been cooled from 300 K down to 2 K, and its magnetic moment has been recorded (ZFC). In the second run, the sample has been brought back to 300 K, a small magnetic field of 100 Oe has been applied parallel to the sample plane and the sample was again cooled down to 2 K; during this cooling procedure, the moment was again recorded (FC). The two runs are depicted in Figure 6. It can be observed that, in ZFC, the variation of the magnetic moment is non-linear; it rises from minimum up to a maximum value of the magnetic moment recorded at a temperature of around 75 K. This temperature is associated to the ferro–superparamagnetic (FM/SPM) transition (T_trans_), thus confirming our assumption made from coercivity vs. temperature dependence (Figure 5).

**Figure 7:**
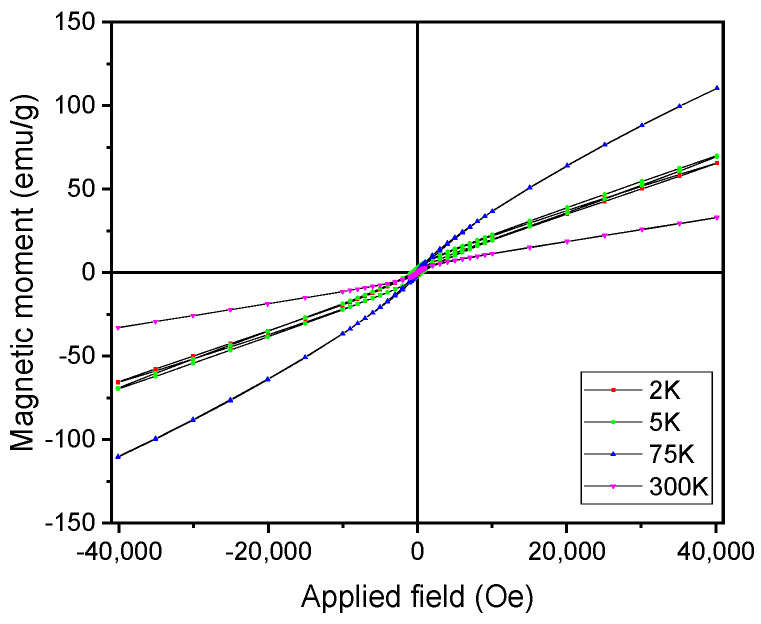
Major hysteresis loops of Mn_52_Al_46_C_2_ alloy at various temperatures.

**Figure 8:**
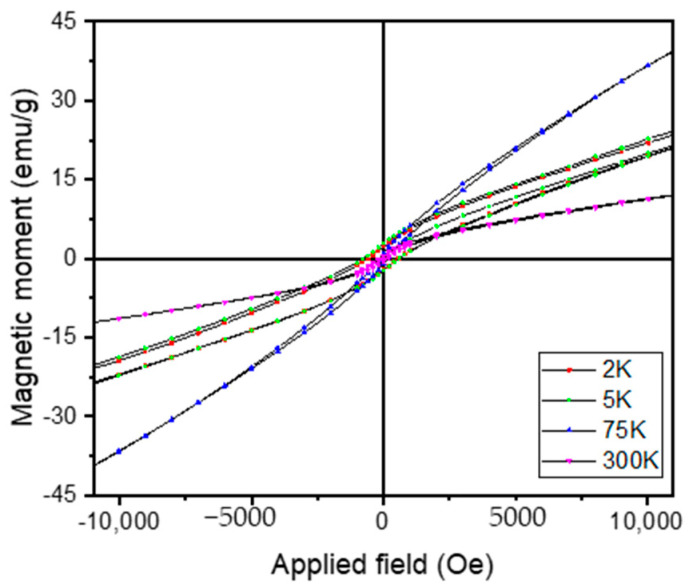
Minor hysteresis loops of Mn_52_Al_46_C_2_ alloy at various temperatures. Coercive fields observed at low T confirm results from Figure 5.

When proceeding to lower temperatures, the magnetic moment decreases; this decrease also shows a kink at a temperature of about 55 K. This kink is associated with a spin reorientation transition (SRT), which is typically observed in magnetic alloys exhibiting FM/SPM magnetic transition. The magnetic moments of individual nanograins are gradually reversed with temperature decrease, and this reversal occurs via two distinct mechanisms: (i) between FM/SPM and SRT there is an in-plane reversal mechanism, with slower variation of the overall magnetic moment; and (ii) between SRT and 2 K there is an out-of-plane reversal mechanism, showing sharper decrease in the overall magnetic moment. This measurement is showing, thus, the various regimes of the magnetic moment: a ferromagnetic state with moments governed by spin reorientation phenomenon, below T_trans_, and a superparamagnetic-like state, above this value, with low magnetization and low coercivity. The FC curve shows quite different behavior. Upon cooling down from 300 K, the magnetic moment slowly increases with an allure rather similar to the one from the ZFC curve, down to T_trans_. The FC curve shows again a maximum of the magnetic moment at this point; however, upon further decrease in temperature, down to 2 K, the magnetic moment does not decrease but rather shows a plateau-like behavior with quasi constant values of magnetic moment. This behavior is quite characteristic for superparamagnetic systems with interacting magnetic grains, where individual magnetic moments fluctuate coherently. Such behavior witnesses the occurrence of a blocking regime, from 75 K down to 2 K, for the MnAl phase. This blocking regime is dictated by the alignment of several of the magnetic nanograins with the aid of the small applied magnetic field, which possesses sufficient magnetic anisotropy energy to overcome the thermal barrier. This blocking regime is typical in systems with magnetic nanograins possessing superparamagnetic behavior. Another magnetic experimental protocol has been performed by measuring major and minor hysteresis loops with the applied field perpendicular on the sample plane for the Mn_52_Al_46_C_2_ alloy. SQUID magnetometry has been again used with a magnetic field of up to 4 Tesla and applied perpendicular to the ribbons; the measurements were taken at temperatures between 2 K and 300 K. Major loops are shown in Figure 7, and minor loops are shown in Figure 8. The magnetization is shown to rise continuously with the applied magnetic field without any tendency for saturation; this is a behavior that is typical for systems in superparamagnetic regime. It is interesting to observe that the magnetization does not saturate even at the highest applied field, but the highest values of the magnetization, in both major and minor loops for high applied fields, are recorded for the sample at 75 K (blue curve).

This confirms the ZFC-FC results where a maximum of the magnetic moment was recorded at the transition temperature of 75 K and illustrates well the transition that was previously evidenced. It is also observed that above this transition, for hysteresis loops recorded at 75 K and 300 K, there is virtually no hysteresis recorded neither in major nor in the minor loops. On the contrary, for temperatures below this transition where the loops were recorded at low temperatures (2 K and 5 K), there is a significant coercive field recorded that is visible mostly in the minor loops from Figure 8. This behavior also confirms well the results depicted in Figure 5 and highlights once again the occurrence of the two regimes of different magnetic behavior in the Mn-Al-C samples below 300 K.

## 4. Discussion

The MnAl system is part of the largest class of magnetic materials with various magnetic states; as a function of temperature, the MnAl system has properties that are highly dependent on synthesis routes. It has been proven that various synthesis routes combined with appropriate annealing can provide various and potentially radically different magnetic parameters [54,55,56,57,58,59,60,61,62,63,64]. Many authors documented [65,66,67,68] that the observed hysteresis in Mn-Al alloys is normally associated with domain wall pinning in antiphase boundaries and stacking faults. Such defects also have a role in creating nucleation sites [66,67] and creating local dislocations [68,69,70,71]. This high level of defects is, however, also induced by the substitution of few atoms of C, and this has been proven to be beneficial in decreasing the ordering temperature and increasing the amount of L1_0_ tetragonal phase at a given stage of annealing. The magnetic system is, however, complicated by the tendency of antiferromagnetic ordering of Mn atoms, therefore making unusual spin structures, with peculiar behavior both at low and high temperatures. The behavior and the stability of the MnAl alloy has been studied and documented at high temperatures by using synchrotron X-ray diffraction. The magnetic structure and the various magnetic regimes of the MnAl alloys, induced by the unusual MnAl spin lattices, has been studied at low temperatures by using complex magnetic measurements. From these measurements, different magnetic regimes were detected and evidenced. Hence, the evolutions of the magnetic behavior and tetragonal phase stability were well documented over a wide range of temperatures by using different and complementary experimental techniques.

It is known that the tetragonal τ phase is formed during a transformation of the hcp ε–phase. Some techniques such as mechanical alloying [7,71,72,73] do not allow the creation of single phase alloys; therefore, obtaining good magnetic properties is still a challenge, even after appropriate annealing. The synthesis technique we choose, being a non-equilibrium technique that ensures alloying in molten state at 1300 °C, ensures high enough τ phase abundance and may result in good magnetic performances.

## 5. Conclusions

We have undertaken a combination of complex experimental protocols, involving both structural, morphologic and magnetic measurements in order to monitor both the stability and the magnetic behavior of Mn-Al-C system during the ε–τ phase transformation over a wide range of temperatures. Two melt spun alloys of compositions Mn_53_Al_45_C_2_ and Mn_52_Al_46_C_2_ were conceived and prepared by melt spinning. The alloys’ morphologies were investigated by SEM imaging, and structural analysis has confirmed that the alloy is single phased and mainly consists of the hexagonal ε phase. The evolution of the structural ε–τ phase transformation has been monitored up to 800 °C by using synchrotron X-ray diffraction, and this technique was proven to be very effective in the estimation of the relative abundance of the tetragonal phase formed during the transformation. Its stability was equally proven to be effective over the large temperature range between 50 °C and 800 °C. For the low temperature regime, complex magnetic measurements, consisting of measuring major and minor loops as well as ZFC-FC magnetization measurements, were employed in order to detect and to demonstrate various magnetic regimes. A spin reorientation transition was proven to occur at 55 K while a maximum magnetization observed in ZFC-FC curves proved that at about 75 K there is a ferro to superparamagnetic state transition. The FC magnetization curve has also proven the existence of a blocking regime, below 55 K, that is characteristic of nanogranular systems with superparamagnetic behavior. The FM/SPM transition was also detected in the hysteresis loops, and higher coercivities are documented for temperatures situated below the transition temperature in the blocking regime of the Mn-Al-C system.

## Data Availability

The data presented in this study are available on request from the corresponding author. The data are not publicly available due to their patenting potential.

## References

[1] Popov V.V., Maccari F., Radulov I.A., Kovalevsky A., Katz-Demyanetz A., Bamberger M. (2021). Microstructure and magnetic properties of Mn-Al-C permanent magnets produced by various techniques. Manuf. Rev..

[2] Thongsamrit W., Charoensuk T., Saetang P., Jantaratana P., Ruttanapun C., Sirisathitkul C. (2021). Effects of Carbon Doping and Annealing Temperature on Magnetic MnAl Powders and MnAl Polymeric Composites. Appl. Sci..

[3] Tyrman M., Ahmim S., Pasko A., Etgens V., Mazaleyrat F., Quetel-Weben S., Perrière L., Guillot I. (2018). Anisotropy of the ferromagnetic L1_0_ phase in the Mn-Al-C alloys induced by high-pressure spark plasma sintering. AIP Adv..

[4] Zeng Q., Baker I., Cui J.B., Yan Z.C. (2007). Structural and magnetic properties of nanostructured Mn–Al–C magnetic materials. J. Magn. Magn. Mater..

[5] Liu Z.W., Chen C., Zheng Z.G., Tan B.H., Ramanujan R.V. (2012). Phase transitions and hard magnetic properties for rapidly solidified MnAl alloys doped with C, B, and rare earth elements. J. Mater. Sci..

[6] Crisan A.D., Vasiliu F., Mercioniu I., Crisan O. (2014). Role of Ag addition in L1(0) ordering of FePt-based nanocomposite magnets. Philos. Mag..

[7] Lee J.G., Wang X.L., Zhang Z.D., Choi C.J. (2011). Effect of mechanical milling and heat treatment on the structure and magnetic properties of gas atomized Mn–Al alloy powders. Thin Solid Films.

[8] Saravanan P., Vinod V.T.P., Černík M., Selvapriya A., Chakravarty D., Kamat S.V. (2015). Processing of Mn–Al nanostructured magnets by spark plasma sintering and subsequent rapid thermal annealing. J. Magn. Magn. Mater..

[9] Coey J.M.D. (2012). Permanent magnets: Plugging the gap. Scr. Mater..

[10] Coey J.M.D. (2014). New permanent magnets; manganese compounds. J. Phys. Condens. Matter.

[11] Umetsu R.Y., Sakuma A., Fukamichi K. (2006). Magnetic anisotropy energy of antiferromagnetic L1_0_-type equiatomic Mn alloys. Appl. Phys. Lett..

[12] Ohtani T., Kato N., Kojima S., Kojima K., Sakamoto Y., Konno I., Tsukahara M., Kubo T. (1977). Magnetic properties of Mn-Al-C permanent magnet alloys. IEEE Trans. Magn..

[13] Kōno H. (1958). On the Ferromagnetic Phase in Manganese-Aluminum System. J. Phys. Soc. Jpn..

[14] Koch A.J.J., Hokkeling P., Steeg M.G.V.D., De Vos K.J. (1960). New Material for Permanent Magnets on a Base of Mn and Al. J. Appl. Phys..

[15] Shukla A., Pelton A.D. (2008). Thermodynamic Assessment of the Al-Mn and Mg-Al-Mn Systems. J. Phase Equilibria Diffus..

[16] Pareti L., Bolzoni F., Leccabue F., Yermakov A. (1986). Magnetic anisotropy of MnAl and MnAlC permanent magnet materials. J. Appl. Phys..

[17] Crisan O., Crisan A.D., Mercioniu I., Nicula R., Vasiliu F. (2016). Development and structural characterization of exchange-spring-like nanomagnets in (Fe, Co)-Pt bulk nanocrystalline alloys. J. Magn. Magn. Mater..

[18] Cui J., Kramer M., Zhou L., Liu F., Gabay A., Hadjipanayis G., Balasubramanian B., Sellmyer D. (2018). Current progress and future challenges in rare-earth-free permanent magnets. Acta Mater..

[19] Crisan O., Crisan A.D., Mercioniu I., Pantelica D., Pantelica A., Vaucher S., Nicula R., Stir M., Vasiliu F. (2015). Effect of Mn addition on the thermal stability and magnetic properties of rapidly-quenched L1_0_ FePt alloys. Intermetallics.

[20] Nicula R., Crisan O., Crisan A.D., Mercioniu I., Stir M., Vasiliu F. (2015). Thermal stability, thermal expansion and grain-growth in exchange-coupled Fe–Pt–Ag–B bulk nanocomposite magnets. J. Alloys Compd..

[21] Crisan A.D., Bednarcik J., Michalik Š., Crisan O. (2014). In situ monitoring of disorder–order A1–L1_0_ FePt phase transformation in nanocomposite FePt-based alloys. J. Alloys Compd..

[22] Crisan A.D., Vasiliu F., Nicula R., Bartha C., Mercioniu I., Crisan O. (2018). Thermodynamic, structural and magnetic studies of phase transformations in MnAl nanocomposite alloys. Mater. Charact..

[23] Müllner P., Bürgler B.E., Heinrich H., Sologubenko A.S., Kostorz G. (2002). Observation of the shear mode of the ε → τ phase transformation in a Mn-Al-C single crystal. Philos. Mag. Lett..

[24] Yanar C., Wiezorek J.M.K., Soffa W.A., Radmilovic V. (2002). Massive transformation and the formation of the ferromagnetic L1_0_ phase in manganese-aluminum-based alloys. Metall. Mater. Trans. A.

[25] Kurtulus Y., Dronskowski R. (2003). Electronic structure, chemical bonding, and spin polarization in ferromagnetic MnAl. J. Solid State Chem..

[26] Sakuma A. (1994). Electronic Structure and Magnetocrystalline Anisotropy Energy of MnAl. J. Phys. Soc. Jpn..

[27] Park J.H., Hong Y.K., Bae S., Lee J.G., Jalli J., Abo G.S., Neveu N., Kim S.G., Choi C.J. (2010). Saturation magnetization and crystalline anisotropy calculations for MnAl permanent magnet. J. Appl. Phys..

[28] Manchanda P., Kashyap A., Shield J., Lewis L., Skomski R. (2014). Magnetic properties of Fe-doped MnAl. J. Magn. Magn. Mater..

[29] Edström A., Chico J., Jakobsson A., Bergman A., Rusz J. (2014). Electronic structure and magnetic properties of L1_0_ binary alloys. Phys. Rev. B.

[30] Houseman E.L., Jakubovics J.P. (1983). Domain structure and magnetization processes in MnAl and MnAlC alloys. J. Magn. Magn. Mater..

[31] Fang H., Cedervall J., Casado F.J.M., Matej Z., Bednarcik J., Ångström J., Berastegui P., Sahlberg M. (2017). Insights into formation and stability of τ-MnAlZ_x_ (Z = C and B). J. Alloys Compd..

[32] Dreizler W., Menth A. (1980). Transformation Kinetics of the Ferromagnetic Alloy Mn-Al-C. IEEE Trans. Magn..

[33] Janotova I., Sr P.S., Svec P., Mat’ko I., Janickovic D., Zigo J., Mihalkovic M., Marcin J., Skorvanek I. (2017). Phase analysis and structure of rapidly quenched Al-Mn systems. J. Alloys Compd..

[34] Lu W., Niu J., Wang T., Xia K., Xiang Z., Song Y., Mi Z., Zhang W., Tian W., Yan Y. (2016). Phase transformation kinetics and microstructural evolution of MnAl permanent magnet alloys. J. Alloys Compd..

[35] Mican S., Benea D., Hirian R., Gavrea R., Isnard O., Pop V., Coldea M. (2016). Structural, electronic and magnetic properties of the Mn_50_Al_46_Ni_4_ alloy. J. Magn. Magn. Mater..

[36] Shao Z., Zhao H., Zeng J., Zhang Y., Yang W., Lai Y., Guo S., Du H., Wang C., Yang Y. (2017). One step preparation of pure τ-MnAl phase with high magnetization using strip casting method. AIP Adv..

[37] Jiménez-Villacorta F., Marion J.L., Oldham J.T., Daniil M., Willard M.A., Lewis L.H. (2014). Magnetism-Structure Correlations during the ε→τ Transformation in Rapidly-Solidified MnAl Nanostructured Alloys. Metals.

[38] Obi O., Burns L., Chen Y., Fitchorov T., Kim S., Hsu K., Heiman D., Lewis L.H., Harris V.G. (2014). Magnetic and structural properties of heat-treated high-moment mechanically alloyed MnAlC powders. J. Alloys Compd..

[39] Fazakas E., Varga L.K., Mazaleyrat F. (2007). Preparation of nanocrystalline Mn–Al–C magnets by melt spinning and subsequent heat treatments. J. Alloys Compd..

[40] Fang H., Kontos S., Ångström J., Cedervall J., Svedlindh P., Gunnarsson K., Sahlberg M. (2016). Directly obtained τ-phase MnAl, a high performance magnetic material for permanent magnets. J. Solid State Chem..

[41] Du Y., Wang J., Zhao J., Schuster J.C., Weitzer F., Schmid-Fetzer R. (2007). Reassessment of the Al-Mn system and a thermodynamic description of the Al-Mg-Mn system. Int. J. Mater. Res..

[42] Mitsui Y., Kobayashi R., Takanaga Y., Takaki A., Umetsu R.Y., Takahashi K., Mizuguchi M., Koyama K. (2019). Different Magnetic Field Effects on the ε−τ Phase Transformation Between (Mn, Zn)–Al and Mn–Al–C. IEEE Trans. Magn..

[43] Dehghan H., Ebrahimi S.A.S. (2019). Effect of hot deformation conditions on magnetic properties of rare earth free magnetic Mn-Al-C alloy. J. Magn. Magn. Mater..

[44] Su K.P., Hu S.L., Wang H.O., Huang S., Chen X.X., Liu J.J., Huo D.X., Ma L., Liu Z.W. (2019). Structural and magnetic properties of Mn_50_Al_46_Cu_4_C_3_ flakes obtained by surfactant-assisted ball milling. Mater. Res. Express.

[45] Kobayashi R., Mitsui Y., Umetsu R.Y., Mizuguchi M., Koyama K. (2021). Synthesis of Ferromagnetic τ-Mn-Al-C by Reactive Sintering. Mater. Trans..

[46] Masrour R., Jabar A., Benyoussef A., Hamedoun M., Bahmad L. (2015). Hysteresis and compensation behaviors of mixed spin-2 and spin-1 hexagonal Ising nanowire core–shell structure. Phys. B Condens. Matter.

[47] Masrour R., Jabar A., Benyoussef A., Hamedoun M. (2018). Magnetic properties of the Ising system on alternate layers of a hexagonal lattice. Phys. A Stat. Mech. Appl..

[48] Crisan A.D., Leca A., Bartha C., Dan I., Crisan O. (2021). Magnetism and ε-τ Phase Transformation in MnAl-Based Nanocomposite Magnets. Nanomaterials.

[49] Klug H.P., Alexander L.E. (1974). X-ray Diffraction Procedures.

[50] Balzar D. (1993). Voigt-function model in diffraction line-broadening analysis. J. Res. Natl. Inst. Stand. Technol..

[51] Balzar D. (1995). BREADTH-a program for analyzing diffraction line broadening. J. Appl. Cryst..

[52] Warren B.E. (1969). X-ray Diffraction.

[53] Gamez J.D., Martínez-Sánchez H., Valenzuela J.L., Marín L., Rodríguez L.A., Snoeck E., Zamora L.E., Alcázar G.A.P., Tabares J.A. (2021). Magnetic τ-MnAlC thin film fabrication by high-vacuum thermal evaporation. Mater. Lett..

[54] Crisan A.D., Nicula R., Crisan O., Burkel E. (2007). Thermally and pressure activated phase evolution in Fe–Pt–Nb–B melt spun ribbons. Mater. Sci. Eng.

[55] Crisan A.D., Crisan O., Randrianantoandro N., Valeanu M., Morariu M., Burkel E. (2007). Crystallization processes in Fe–Pt–Nb–B melt spun ribbons. Mater. Sci. Eng C.

[56] Von Haeften K., Binns C., Brewer A., Crisan O., Howes P.B., Lowe M.P., Sibbley-Allen C., Thornton S.C. (2009). A novel approach towards the production of luminescent silicon nanoparticles: Sputtering, gas aggregation and co-deposition with H_2_O. Eur. Phys. J. D.

[57] Crisan A.D., Crisan O. (2011). Direct formation of L1_0_ FePt in as-cast FePt-based magnetic nanocomposite ribbons without post-synthesis annealing. J. Phys. D Appl. Phys..

[58] Crisan O., Le Breton J.M., Jianu A., Teillet J., Filoti G. (1997). Structural properties of amorphous and nanocrystallized Fe-Cu-Nb-Si-B and Fe-Gd-Cu-Nb-Si-B ribbons. J. Alloys Compd..

[59] Rosenberg M., Kuncser V., Crisan O., Hernando A., Navarro E., Filoti G. (1998). A Mossbauer spectroscopy and magnetic study of FeRh. J. Magn. Magn. Mater..

[60] Crisan O., Labaye Y., Berger L., Coey J.M.D., Greneche J.M. (2002). Exchange coupling effects in nanocrystalline alloys studied by Monte Carlo simulation. J. Appl. Phys..

[61] Crisan O., Crisan A.D., Randrianantoandro N., Nicula R., Burkel E. (2007). Crystallization processes and phase evolution in amorphous Fe–Pt–Nb–B alloys. J. Alloys Compd..

[62] Crisan O., Angelakeris M., Flevaris N.K., Filoti G. (2003). Magnetism and Anisotropy in Core-Shell Nanoparticles. J. Optoelectron. Adv. Mater..

[63] Crisan O., Greneche J.M., le Breton J.M., Crisan A.D., Labaye Y., Berger L., Filoti G. (2003). Magnetism of nanocrystalline Finemet alloy: Experiment and simulation. Eur. Phys. J. B.

[64] McCurrie R., Rickman J., Dunk P., Hawkridge D. (1978). Dependence of the permanent magnet properties of Mn_55_Al_45_ on particle size. IEEE Trans. Magn..

[65] Zijlstra H. (1970). Coercivity and wall motion. IEEE Trans. Magn..

[66] Jakubovics J., Jolly T. (1977). The effect of crystal defects on the domain structure of Mn-Al alloys. Phys. B + C.

[67] Bance S., Bittner F., Woodcock T., Schultz L., Schrefl T. (2017). Role of twin and anti-phase defects in MnAl permanent magnets. Acta Mater..

[68] Bittner F., Freudenberger J., Schultz L., Woodcock T. (2017). The impact of dislocations on coercivity in L1_0_-MnAl. J. Alloys Compd..

[69] Thielsch J., Bittner F., Woodcock T.G. (2017). Magnetization reversal processes in hot-extruded τ-MnAl-C. J. Magn. Magn Mater..

[70] Livingston J.D. (1981). A review of coercivity mechanisms (invited). J. Appl. Phys..

[71] Lucis M.J., Prost T.E., Jiang X., Wang M., Shield J.E. (2014). Phase Transitions in Mechanically Milled Mn-Al-C Permanent Magnets. Metals.

[72] Jian H., Skokov K.P., Gutfleisch O. (2015). Microstructure and magnetic properties of Mn–Al–C alloy powders prepared by ball milling. J. Alloys Compd..

[73] Bittner F., Schultz L., Woodcock T. (2017). The role of the interface distribution in the decomposition of metastable L1_0_-Mn_54_Al_46_. J. Alloys Compd..

